# Contemporary point of care cardiac troponin testing in suspected acute coronary syndrome

**DOI:** 10.1136/heartjnl-2018-314306

**Published:** 2019-02-07

**Authors:** Andrew R Chapman, Stacey Stewart, Nicholas L Mills

**Affiliations:** 1 BHF Centre for Cardiovascular Science, University of Edinburgh, Edinburgh, UK; 2 Usher Institute of Population Health Sciences and Informatics, The University of Edinburgh, Edinburgh, UK

**Keywords:** acute myocardial infarction

Around one million patients present to hospital with chest pain every year, accounting for approximately 5% of all emergency department attendances in the UK. However, only one in five patients are found to have had a myocardial infarction.^1^ Therefore, for several years, there has been a drive to develop diagnostic strategies which allow accurate identification of patients without myocardial infarction at an earlier stage, who may not require admission to hospital for serial cardiac biomarker testing. Such strategies have the potential to improve patient experience and optimise resource allocation both in the emergency department and in hospital, at a time of ever-increasing demands.

Cardiac troponin is the biomarker of choice for the detection of myocardial injury, and international guidelines recommend concentrations are measured using a high-sensitivity assay.^2^ While high-sensitivity assays were first introduced in Europe in 2010, they have only recently become available for use in clinical practice in the USA. The higher precision and lower limits of detection afforded by these tests has facilitated the development of pathways which can rule out myocardial infarction at an earlier stage, the majority of which have demonstrated a magnitude of benefits in diagnostic accuracy compared with using the recommended diagnostic threshold (99th centile) alone.^3 4^ In practical terms, implementation of these approaches requires investment in infrastructure to deliver accurate and timely cardiac troponin results on a high-sensitivity platform which is not always available.

**Figure 1:**
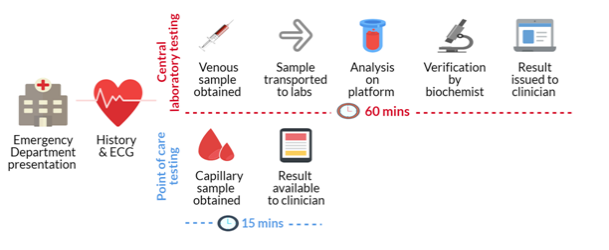
Point of care compared with central laboratory testing for cardiac troponin in patients with suspected acute coronary syndrome.

One potential strategy to improve efficiency is through the use of point of care devices. Similar to blood glucose testing, the delivery of rapid and accurate measurements of cardiac troponin at the bedside could allow earlier diagnosis or rule out of myocardial infarction in practice see figure 1. Body *et al* report results from a prospective cohort study evaluating the diagnostic accuracy of the Troponin-only Manchester Acute Coronary Syndrome (T-MACS) decision aid when applied using a contemporary point of care cardiac troponin I assay. The Abbott iSTAT point of care assay has a reported 99th centile of 80 ng/L and limit of detection of 20 ng/L, with a coefficient of variation of 16.5% at the 99th centile diagnostic threshold.^5^ In this study, cardiac troponin I concentrations were measured on a central laboratory platform and on the point of care device in 716 patients across eight sites in England. When T-MACS was applied with the 99th centile of the iSTAT assay at 0 and 3 hours, the authors report excellent diagnostic accuracy, with an Negative predictive value (NPV) of 99.5% (95% CI 96.5% to 99.9%) and sensitivity of 99.0% (95% CI 94.4% to 100%), for a primary outcome of myocardial infarction, coronary revascularisation or all-cause death at 30 days.

While the findings of Body *et al* are encouraging, there are important limitations to consider. First, the majority of patients were male (74.3%), and only those with symptoms for less than 12 hours were included. Although this may reduce the generalisability of the findings, focusing on patients with a shorter duration of symptoms would be expected to reduce diagnostic accuracy, as troponin release is a time-dependent phenomenon. Blood samples were obtained at the bedside but were processed by research nurses not involved in patient care, so the impact on diagnostic efficiency is unmeasurable. Finally, in keeping with the majority of studies in this area, the results are based on observational data. Patients were not managed on the basis of test results and may have undergone additional investigations and management which could have influenced their outcomes. Importantly, local validation of this approach is essential to adjust for differences in population level characteristics and the pretest probability of disease which may influence diagnostic performance.

It should be acknowledged that it is difficult to conduct observational cohort studies in patients with suspected acute coronary syndrome that replicate real world clinical practice. Indeed, in the majority of studies evaluating cardiac troponin use, the symptom onset to sample time is around 6 hours. With the availability of bedside testing, this time may significantly fall and diagnostic performance may be less robust. Furthermore, the availability of easily accessible bedside troponin testing may lead to less selective, non-judicious testing which could lead to an increase in the diagnosis of type 2 myocardial infarction or myocardial injury.^6^

Although the focus of the study of Body *et al* is on the in-hospital use of point of care testing, there are a number of novel approaches which could be transformative for clinical practice. Prehospital use may facilitate the evaluation of suspected myocardial infarction in the community, allowing redirection of patients with a higher probability of myocardial infarction to cardiac centres, and low risk patients to district general hospitals. The Pre-hospital Evaluation of Sensitive Troponin study will evaluate the performance of pre-hospital cardiac troponin testing (NCT:03561051). The study aims to recruit 700 patients who have phoned the emergency services with symptoms suspicious for acute coronary syndrome *before* they arrive at hospital. Blood samples will be obtained in the ambulance and on arrival to hospital. While tests will not be run live, this study will obtain samples as near to symptom onset as practicable, giving important insight into the diagnostic accuracy of point of care testing and allowing for validation of the T-MACS clinical risk score in this population.

In future, the diagnostic performance of point of care cardiac troponin assays is likely to improve. Recently, a novel point of care cardiac troponin I assay was described by Pickering *et al* in a pilot study of 354 patients. While not formally designated as a high-sensitivity assay, the Abbott TnI-Nx is capable of reporting concentrations from 1 to 1500 ng/L.^7^ In their evaluation, they found this assay could rule out myocardial infarction on the basis of a single troponin result, with comparable NPV and sensitivity to the established ARCHITECT*_STAT_* high-sensitivity cardiac troponin I laboratory platform. Whether the addition of this novel assay to the T-MACS strategy could allow more patients to be identified as low risk in the emergency department remains unclear, but would be an important evaluation in future.

Contemporary point of care cardiac troponin testing in combination with a clinical risk score may facilitate the rule-out of myocardial infarction in institutions without access to a high-sensitivity cardiac troponin platform. While novel, more sensitive point of care assays are in development, the encouraging findings of Body *et al* should lead to prospective validation studies to demonstrate the safety and efficacy of this approach.
